# Photoelectron diffraction from laser-aligned molecules with X-ray free-electron laser pulses

**DOI:** 10.1038/srep14065

**Published:** 2015-09-15

**Authors:** Kyo Nakajima, Takahiro Teramoto, Hiroshi Akagi, Takashi Fujikawa, Takuya Majima, Shinichirou Minemoto, Kanade Ogawa, Hirofumi Sakai, Tadashi Togashi, Kensuke Tono, Shota Tsuru, Ken Wada, Makina Yabashi, Akira Yagishita

**Affiliations:** 1 Institute of Materials Structure Science, KEK, 1-1 Oho, Tsukuba, Ibaraki 305-0801, Japan; 2 College of Science and Engineering, Ritsumeikan University, 1-1-1 Noji-higashi, Kusatsu, Shiga 525-8577, Japan; 3 Quantum Beam Science Center, Japan Atomic Energy Agency, 8-1-7 Umemidai, Kizugawa, Kyoto 619-0215, Japan; 4 Graduate School of Science, Chiba University, 1-33 Yayoi-cho, Inage-ku, Chiba 263-8522, Japan; 5 Quantum Science and Engineering Center, Kyoto University, Uji, Kyoto 611-0011, Japan; 6 Department of Physics, Graduate School of Science, The University of Tokyo, 7-3-1 Hongo, Bunkyo-ku, Tokyo 113-0033, Japan; 7 RIKEN SPring-8 Center, 1-1-1 Kouto, Sayo-cho, Sayo-gun, Hyogo 679-5148, Japan; 8 Japan Synchrotron Radiation Research Institute, 1-1-1 Kouto, Sayo-cho, Sayo-gun, Hyogo 679-5198, Japan

## Abstract

We report on the measurement of deep inner-shell 2*p* X-ray photoelectron diffraction (XPD) patterns from laser-aligned I_2_ molecules using X-ray free-electron laser (XFEL) pulses. The XPD patterns of the I_2_ molecules, aligned parallel to the polarization vector of the XFEL, were well matched with our theoretical calculations. Further, we propose a criterion for applying our molecular-structure-determination methodology to the experimental XPD data. In turn, we have demonstrated that this approach is a significant step toward the time-resolved imaging of molecular structures.

X-ray free-electron lasers (XFELs) are expected to permit high-resolution, femtosecond coherent X-ray diffractive imaging of nanometer- to micrometer-sized objects without requiring their crystalline periodicity^1^,^2^. Reconstructed images are directly obtained from coherent X-ray diffraction patterns by phase retrieval through an oversampling method^3^,^4^,^5^,^6^. Such promising concepts with XFELs have been demonstrated successfully^7^,^8^,^9^,^10^. For isolated gas-phase molecules, the first attempt of X-ray diffraction measurements with XFEL was reported very recently, employing a technique of aligning molecules with strong laser pulses^11^. However, these measurements suffered from very low X-ray elastic-scattering cross sections, making it difficult to acquire good signal-to-noise ratios.

As an alternative to X-ray diffraction, photoelectron holography using XFEL was proposed by a German group^12^. Then, we have recently proposed a more practical scheme of photoelectron diffraction to capture molecular movies on a femtosecond scale with Ångström spatial resolution for small- to medium-sized gas-phase molecules^13^. This method relies on the capabilities of XFEL, velocity-map imaging of photoelectrons^14^, and control of molecular alignment by the strong electric fields of optical lasers^15^,^16^,^17^, and is based on a well-developed X-ray photoelectron diffraction (XPD) scenario for surface-structure analysis^18^,^19^,^20^,^21^. The first attempt of such XPD measurements was performed recently at the Linac Coherent Light Source (LCLS)^22^,^23^. Despite these efforts, these measurements suffered from cluster contamination in sample molecules, making it difficult to obtain XPD data of the laser-aligned molecules. Thus, the differences between the XPD data with and without alignment laser have been examined. The difference data manifesting interference between photoelectron partial-waves have been well reproduced by theoretical calculations. Although these results may be promising, XPD data of the laser-aligned target molecules are desirable for their structure determination.

In this Article, we report on I 2*p* photoelectron angular distributions from both randomly oriented and laser-aligned I_2_ molecules using XFEL pulses from the SPring-8 Ångström Compact free-electron LAser (SACLA). We selected a kinetic energy of 140 eV for the 2*p* photoelectrons, which ensures that in an aligned molecule the XPD pattern is dominated by a photoelectron wave emitted from an emitter atom and a scattered wave from a neighboring atom, thus exhibiting the interference structure between the two electron waves (see Fig. 1)^13^,^24^,^25^,^26^. This situation, in which two electron waves interfere depending on both the internuclear distance and electron energy (in other words, the electron de Broglie wavelength), is similar to that in Young’s double-slit experiments. In fact, the measured XPD patterns are well reproduced by XPD theory. We then demonstrate a criterion for applying our molecular-structure-determination methodology^13^ to the experimental data. Consequently, we have confirmed that this approach is a critical step toward the time-resolved imaging of molecular structures.

## Results

### Concepts for the experimental set-up

**Figure 2:**
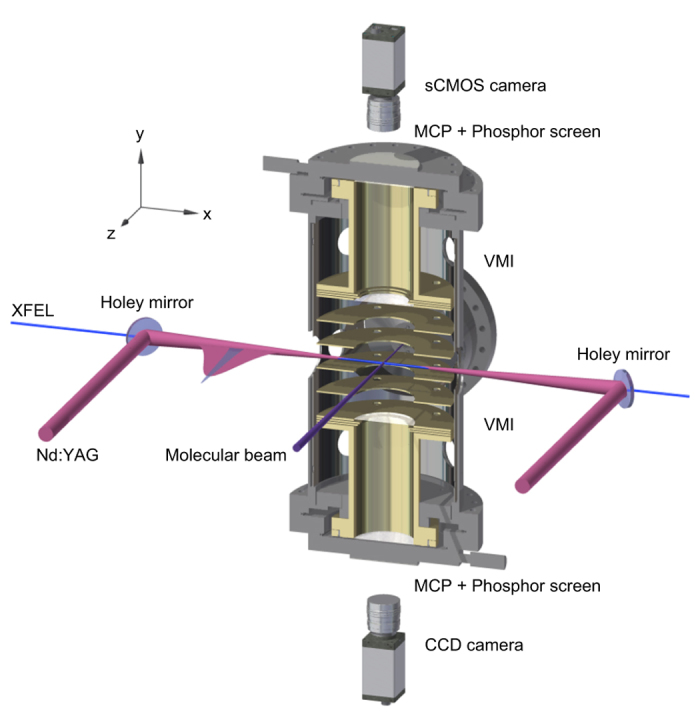
Schematic drawing of the experimental set-up. Two laser beams propagating in a collinear arrangement intersect a supersonic pulsed molecular beam at the center of a vacuum chamber. A Nd:YAG laser is used to align the sample molecules probed by the XFEL. XPD images of photoelectrons are recorded by the upper VMI. The degree of alignment is quantified using 2D momentum distributions of ionic fragments, which are registered by the lower VMI.

Our experimental apparatus consists of facing velocity-map imaging spectrometers (VMIs), supersonic pulsed molecular beams, and a Nd:YAG laser for molecular alignment (see Fig. 2)^13^. Between the facing VMIs, the pulsed molecular beams are crossed by collinear pulsed lasers (Nd:YAG laser and XFEL). Then, electrons produced in the interaction region are drawn into the upper VMI, while ions are drawn into the lower VMI: the former records two-dimensional (2D) photoelectron diffraction images, and the latter records 2D fragment-ion images to monitor the degree of alignment of the I_2_ molecules in real time.

The apparatus was attached to the beamline BL3 in the experimental hutch EH3 at SACLA^27^,^28^, and our measurements were taken at the end-station. XPD patterns of photoelectron energies between 100 and 200 eV are preferable for our molecular-structure-determination methodology^13^. Therefore, we selected a mean photon energy of 4697 eV for the probing XFEL pulses, which is above the I_3/2_ ionization threshold of 4557 eV. This creates I 2*p* photoelectrons with a mean energy of 140 eV, although the photon energies of the XFEL fluctuate within a band-pass range of 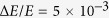 shot-by-shot^28^. We neglect the interaction between the spin and the orbital motion of each electron hereafter. For further reference, the experimental details are described in the Methods section.

### Photoemission from laser-aligned molecules

**Figure 3:**
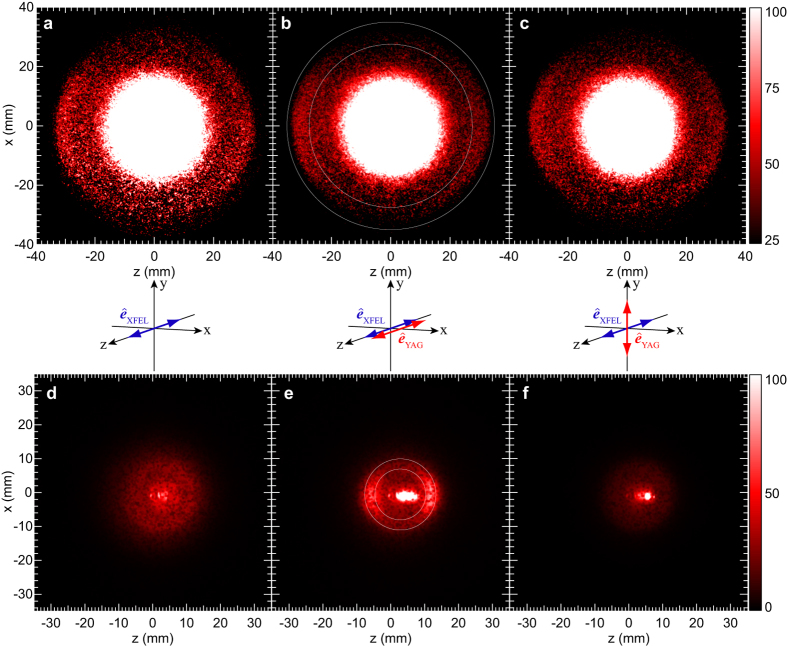
2D momentum images of electrons and ions. Outer rings in (**a**–**c**) are due to I 2*p* photoelectrons. Rings in (**d**–**f**) are due to fragment ions I^*m*+^, see Methods. Centers of the rings shift toward the down stream direction, +*z*, of the molecular beams. Three sets of (**a**,**d**), (**b**,**e**), and (**c**,**f**) were measured simultaneously. Insets in the middle row indicate polarization geometries. Polarization vectors of the Nd:YAG laser and XFEL are indicated by double-headed arrows. In (**b**), the photoelectron image bounded by white circles with the radii of 27.5 and 35 mm is distingushed from the central-ring image of low-energy electrons. In (**e**), the fragment-ion image bounded by white circles with the radii of 7.5 and 10.5 mm indicates that molecular axis distributions are aligned along the polarization vector of the Nd:YAG laser.

2*p* photoelectron momentum images and fragment-ion momentum images of the I_2_ molecules under different experimental conditions are shown in Fig. 3, along with illustrations of relevant polarization geometries. Each image was obtained from alternating measurements of the images with and without the I_2_ molecular beams, which was operated at half of the XFEL repetition rate.

The 2D momentum images of the fragment ions created by the probing XFEL (bottom row) provide the basic experimental information. The observation of circularly symmetric rings, in Fig. 3(d), shows that the initial orientation of the molecules is random. The angular distribution of I^*m*+^ fragment ions, with 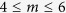, is localized around the polarization direction of the Nd:YAG laser, see Fig. 3(e). This is an effect of the molecules being aligned by the laser pulse along its field vector. From the angular distribution of a prominent ring bounded by the radii *r* = 7.5 mm and 10.5 mm, which originates from the Coulomb explosion of fragment-ion pairs of I^*m*+^ and I^*n*+^ ions, with 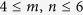, we determined the degree of alignment^16^ of 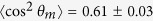, where 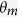 is the angle between the polarization vector and the molecular axis being parallel to fragment-ion momentum direction (see Methods). In Fig. 3(f), the prominent ring in Fig. 3(e) nearly disappears, indicating that there are few molecules left in the xz plane, which is parallel to the detector surface. Thus, the observed results for the fragment ions ensure that the photoelectron-momentum image in Fig. 3(a) comes from the randomly oriented molecules, and that the images in Fig. 3(b,c) are from the aligned molecules.

On this basis, we turn to a qualitative examination of the momentum images of the 2*p* photoelectrons. The electron momentum images in Fig. 3(a–c) in the upper row are composed of the extremely strong central ring and the outer ring of the 2*p* photoelectrons. In deep inner-shell photoionization, as with I 2*p*, great quantities of low-energy electrons are produced frequently via shake-off processes induced by Auger transitions, so that intense signals appear in the central region of the VMI detector. This condition does apply for our experiment; however, thanks to the high photoelectron energy (140 eV), the outer-ring photoelectron image (between *r* = 27.5 and 35 mm) is distinguished from the central-ring image of low-energy electrons, although the outer-ring image is blurred by the photon-energy shot-by-shot fluctuation, 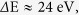 and by the azimuthal-angle distribution. Nevertheless, one can differentiate between the outer-ring images in Fig. 3(a,b), without and with the alignment laser, respectively. This difference is minor but clearly visible: the lengths of the bright arc segments in Fig. 3(b) are slightly shorter than those in Fig. 3(a). It should be noted that the expected interference structure in Fig. 3(b) may be smeared out by molecular-axis distributions, because the degree of alignment in this experiment is not sufficient,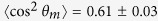, which means that about 60% of all the molecules have their axis located within a cone of 40°.

**Figure 4:**
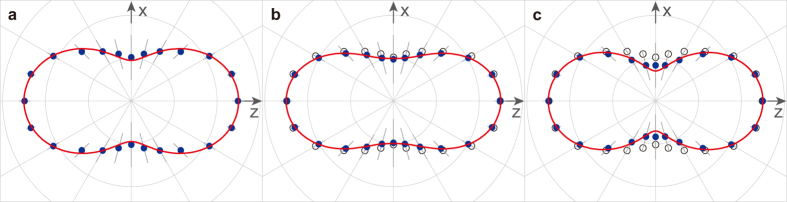
Polar plots of I 2*p* photoelectron angular distributions. (**a**–**c**) were constructed from the photoelectron images in Fig. 3(a–c), respectively. Filled circles with error bars represent the experimental data, and solid curves are fitted results. The errors did not have a normal distribution. In the figures, the maximum values are normalized. To see the small differences in the figures, the data in (**a**), open circles, are superimposed in (**b**,**c**). For details, see Methods.

For further quantitative discussion on the photoelectron angular distributions measured by the momentum images in Fig. 3, polar plots are provided in Fig. 4. Data processing for the polar plots are written in the Methods section. Although the differences in Fig. 4(a–c) are small, one can recognize them in the polar plots. The reference frame of Fig. 4(a) is the laboratory coordinate system, whose *z* axis is the direction of XFEL’s polarization vector. In this frame, the angular distribution is expressed by Eq. (1) in the Methods section. Thus we fitted Eq. (1) to the experimental data and calculated the asymmetry parameter β to be 0.65 ± 0.12. On the other hand, the photoelectron angular distribution in Fig. 4(b) is presented in the molecular reference frame, whose *z* axis is the molecular axis parallel to the direction of the polarization vector of the alignment laser (see Fig. 3). In the molecular frame, the photoelectron angular distribution is determined by Eq. (2) in the Methods section. Such angular distribution is equivalent to the XPD pattern, so that hereafter it is referred to as XPD. Even if the molecular axes are not fully aligned along the *z* axis (parallel to the polarization vector), the functional form of Eq. (2) is applicable to reproduce the experimental results. Therefore, we fitted Eq. (2) with up to *L* = 6 to the experimental data. With the help of this theoretical analysis of the numerical data, it is highlighted that the XPD pattern in Fig. 4(b) clearly exhibits a different shape from that for the photoelectron angular distribution in Fig. 4(a). Besides such a visual check, we examined the difference between the shapes shown in Fig. 4(a,b) quantitatively: Eq. (1) was fitted to the data in Fig. 4(b) and Eq. (2) to the data in Fig. 4(a). A weighted sum of squared errors for the each least squares fitting is summarized in Table 1. From this, one can see that Eq. (1) gives the smaller minimum weighted sum for Fig. 4(a) than that for Fig. 4(b) and that Eq. (2) gives the smaller minimum weighted sum for Fig. 4(b) than that for Fig. 4(a). These results are the firm evidence that the shape of the angular distribution shown in Fig. 4(b) differs from that in Fig. 4(a). Here we emphasize that the comparison of the minimum weighted sums for Eq. (1) and Eq. (2) about one data (Fig. 4(a) or Fig. 4(b)) does not make sense because the mathematical forms of them are different from each other. The comparison of the diffraction pattern with theoretical XPD results will be discussed later.

In the molecular frame, the XPD pattern in Fig. 4(c) is fitted with Eq. (3) in the Methods section. In this case, the molecular axis is aligned along the *y* axis in the figure (see Fig. 3) perpendicular to the *xz* plane of the paper, so that the polar plot gives the azimuthal-angle distribution of the XPD pattern. As can be seen from Eq. (3), the characteristic features of the azimuthal-angle distribution are restricted by conservation of angular momentum component on the molecular axis, so that the XPD pattern observed in this perpendicular polarization geometry provides less information about the molecular geometry than that in the parallel polarization.

### Simulated XPD patterns

**Figure 5:**
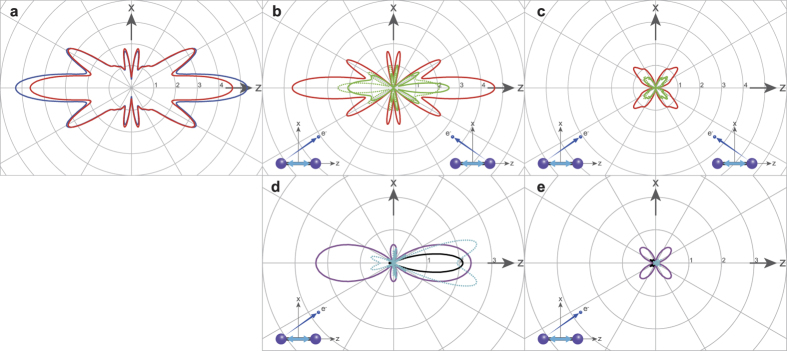
Calculated I 2*p* XPD patterns. (**a**) Blue curve: full multiple-scattering calculation; red curve: single-scattering calculation. (**b**) The green bold and dotted curves are the 2*p*_*z*_ XPD patterns from left-side and right-side I atoms under the single-scattering approximation, respectively. The red curve is an incoherent superposition of the two XPD patterns. (**c**) Same as (**b**) but for the 2*p*_*x*_ XPD pattern. (**d**) Purple curve: primary photoemission amplitude from the 2*p*_*z*_ in the left-side I atom, 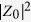, black curve: single-scattering amplitude, 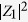, and light-blue curve: interference term of 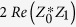 (with positive values expressed by the bold curve and negative values by the dotted curve). (**e**) Same as (**d**) but for the 2*p*_*x*_. In (**e**) the black curve for 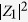 and the light-blue curve for 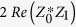 are barely visible. Insets show the polarization geometry, in which a double-headed arrow indicates the polarization vector of the XFEL.

We now discuss how the multiple-scattering XPD theory helps us to interpret the observed XPD results (detailed in the Methods section). The 2*p* state is triply degenerate, so we consider the photoemission from the 2*p*_*z*_ orbital to be aligned along the molecular axis and from the 2*p*_*x*_ (2*p*_*y*_) orbital to be aligned along the *x* axis (*y* axis) orthogonal to the molecular axis. The theoretical results for the XPD, which were calculated for light polarization along the molecular axis, are depicted as polar plots on the *xz* plane in Fig. 5 [i.e., XPD from the 2*p*_*z*_ orbital: Fig. 5(b), that from the *2p*_*x*_ orbital: Fig. 5(c), and their sum: Fig. 5(a)]. Here, we take incoherent superposition of XPD from both the left and right I atoms. As can be seen in Fig. 5(a), the difference between XPD by full multiple-scattering calculation and that by single-scattering calculation is minor. This implies that at the photoelectron energy of 140 eV, the single-scattering effect predominates in XPD, as reported in the literature^13^,^24^,^25^,^26^.

To elucidate the interference effect in XPD, the results of the computational experiment for the single-scattering approximation, 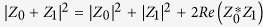, are shown in Fig. 5(d,e) (see Methods). For the polarization geometry corresponding to light polarized along the molecular axis, the photoionization of the 2*p*_*z*_ orbital creates both *s*- and 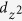-partial waves in the local region of the emitter’s atomic site, owing to the dipole selection rule. In Fig. 5(d), however, the primary photoemission amplitude, 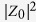, exhibits the specific shape of the angular function of 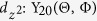, where 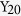 is a spherical harmonic, because the minor component of the *s-*partial wave contributes negligibly. Since the neighboring atom lies in the preferential direction of primary photoemission, the appreciable amplitude,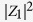, of the wave scattered by the atom is observed in the forward direction. Thus, one can expect a strong interference effect,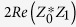, between the primary photoelectron and scattered waves. In fact, 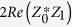 strongly modulates the shape of 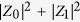, see Fig. 5(b,d). On the other hand, the photoionization of the 2*p*_*x*_ orbital produces a *d*_*xz*_ *-*partial wave at the emitter’s atomic site. This is made obvious by Fig. 5(e): the primary photoemission amplitude, 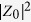, exhibits the characteristic shape of the angular function of 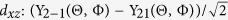. In this case, since the neighboring atom resides on the angular node of 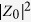, the amplitude, 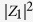, of the wave scattered by this atom is quite small: it is barely visible in Fig. 5(e). Consequently, the interference effect,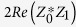, between the primary photoelectron and scattered waves appears weakly in the *2p*_*x*_ photoionization, see Fig. 5(c,e).

### Computational and experimental results

**Figure 6:**
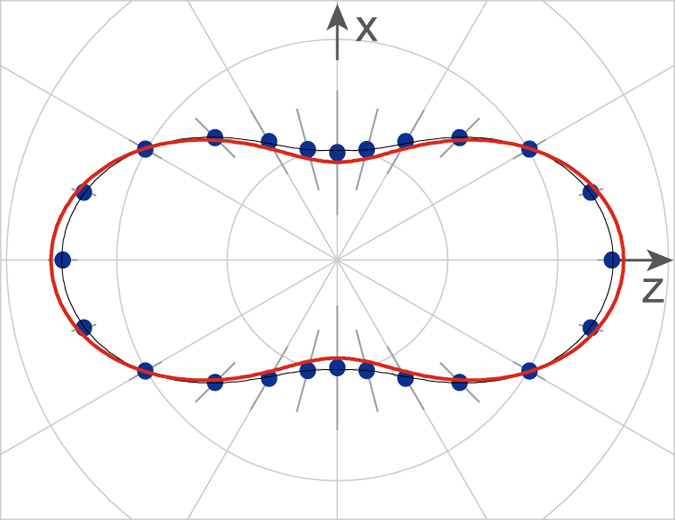
Comparison of computational and experimental I 2*p* XPD patterns. Bold solid curve: full multiple-scattering calculation taking the molecular axis distribution into account. Filled circles with error bars: experimental data. Thin solid curve: fitted result. The experimental data are the same as in Fig. 4(b). Each result was normalized by the area of the polar plots.

Here, we build on the two preceding subsections by comparing the observed and theoretical XPD patterns. The interference fine structure of the XPD shown in Fig. 5(a) is not observed in the XPD shown in Fig. 4(b). As mentioned earlier, this occurs because the molecular axes of I_2_ are not fully aligned in our experiments. Thus, the axis distribution expressed by 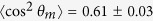 was taken into account in the XPD calculations. The acceptance angle, when we made the polar plot in Fig. 4(b), was also taken into account in the calculations. The computational results by the multiple-scattering XPD theory are shown in Fig. 6, along with the experimental results. In these calculations, we used an equilibrium internuclear distance of 2.666 Å for the ground-state I_2_ molecule, which is the sole geometrical parameter in this practical application. As seen in Fig. 6, the theoretical results taking the axis distribution into account reproduce the experimental ones quite well. This demonstrates that the multiple-scattering XPD theory is a promising computational means for deriving molecular structures from experimental XPD patterns, which are more or less influenced by axis distributions of sample molecules.

## Discussion

**Figure 7:**
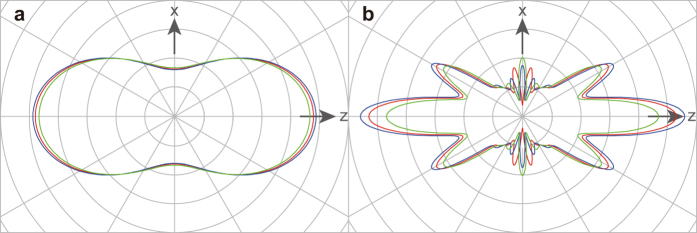
I 2*p* XPD patterns depending on internuclear distances. (**a**) Patterns for 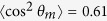, and (**b**) for 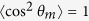. Red curves: equilibrium internuclear distance of 2.666 Å; blue curves: internuclear distance of (2.666 + 0.5) Å; and green curves: internuclear distance of (2.666–0.5) Å. The red curve in (**a**) is the same as the bold solid curve in Fig. 6.

The present XPD pattern is strongly affected by the degree of alignment for the sample molecules, as described above. Thus, a question arises: how sensitive to the molecular structure is the XPD pattern, when averaged by the molecular-axis distribution? To answer this question, computational experiments have been performed: we calculated the XPD patterns by changing internuclear distances for both partially and fully aligned I_2_ molecules. These results are shown in Fig. 7. On one hand, the XPD patterns averaged by the axis distribution expressed by 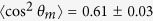 are not especially sensitive to changes in internuclear distance of ±0.5 Å [see Fig. 7(a)]. On the other hand, the XPD patterns from the fully aligned molecules are sensitive to such small changes in the internuclear distance [see Fig. 7(b)]. From this, one can conclude that to definitively determine a molecular structure from a measured XPD pattern, a higher degree of alignment of sample molecules is necessary. In other words, the XPD patterns exhibiting interference profiles, which would be measurable for highly aligned molecules, are essential for the application of our molecular-structure-determination methodology, see Ref. 13. Namely, this is a criterion for the necessary experimental data to derive molecular structures from them. It should be noted that to achieve higher degrees of alignment, for example, an electrostatic-molecular-deflector for selecting quantum states of sample molecules may be a suitable device^17^,^29^,^30^. Further, the most advanced molecular alignment or orientation technique allows us to align or orient state-selected asymmetric top molecules even in the field-free condition^31^.

In conclusion, we have successfully measured 2*p* XPD patterns from laser-aligned I_2_ molecules using XFEL pulses, which were in strong agreement with the multiple-scattering XPD theory calculations. In light of this, we have proposed the criterion for applying our molecular-structure-determination methodology to experimental XPD data. Thus, the present work is a step toward ultra-fast photoelectron diffraction, which may enable the capture of ultra-fast molecular movies, for example, of photochemical reactions.

## Methods

### Experimental details

XFEL pulses with a duration of ~10 fs (FWHM) were triggered at a repetition rate of 30 Hz^27^,^28^. A focal spot size of ~1 μm in diameter was created with a pair of Kirkpatrick-Baez (KB) mirrors^32^. During our experiment, the pulse energy of the XFEL was 300–450 μJ. The Nd:YAG laser pulses, with a duration of ~10 ns (FWHM), a pulse energy of ~800 mJ, and a repetition rate of 30 Hz, were used to align the I_2_ molecules. The Nd:YAG laser was focused to a spot size of 80 μm in diameter at the interaction point, resulting in an intensity of ~10^12^ Wcm^−2^. A spatial overlap between the XFEL and Nd:YAG laser was confirmed by monitoring their spot images on a Ce:YAG screen installed at the interaction region. A temporal overlap between the XFEL and YAG laser pulses was introduced by adjusting the time delay of the Nd:YAG laser pulses to the XFEL pulses, which were measured by a pin photo-diode for the former and a home-made photo-diode for the latter and placed equidistant from the interaction point.

A pulsed supersonic beam of rotationally cold I_2_ molecules was formed by expanding a mixture of I_2_ and He carrier gas from an Even-Lavie valve^33^ with a nozzle diameter of 150 μm, which was then collimated by a skimmer with a diameter of 3 mm. The valve was operated with a duration of 20.7 μs for a driving pulse. The stagnation pressure of He was 35 bar, and the sample pressure of I_2_ gas was ~0.006 bar, which was evaporated from solid I_2_ by heating the valve containing it to 60 °C. Operating the pulse valve at the half of the XFEL repetition rate results in alternating measurements of electron and ion images with and without the sample gas.

The static electric fields in the extraction regions and the drift regions of the VMI were adjusted to optimize the velocity focusing to I 2*p* photoelectrons with a kinetic energy of 140 eV. The VMI was equipped with a micro-channel plate (MCP) backed by a phosphor screen with an active diameter of 75 mm. Electron images were recorded with a scientific complementary metal-oxide-semiconductor (sCMOS) camera mounted to the upper VMI, while ion images were recorded with a CCD camera mounted to the lower VMI. We accumulated momentum-image data for 740,000 XFEL pulses without Nd:YAG laser pulses, for 1,150,000 XFEL pulses with horizontally polarized Nd:YAG laser pulses, and for 550,000 XFEL pulses with vertically polarized Nd:YAG pulses, which correspond to 7 hours, 11 hours, and 5 hours of total time, respectively.

### Fragmentation dynamics upon *2p* ionization of I_2_

**Figure 8:**
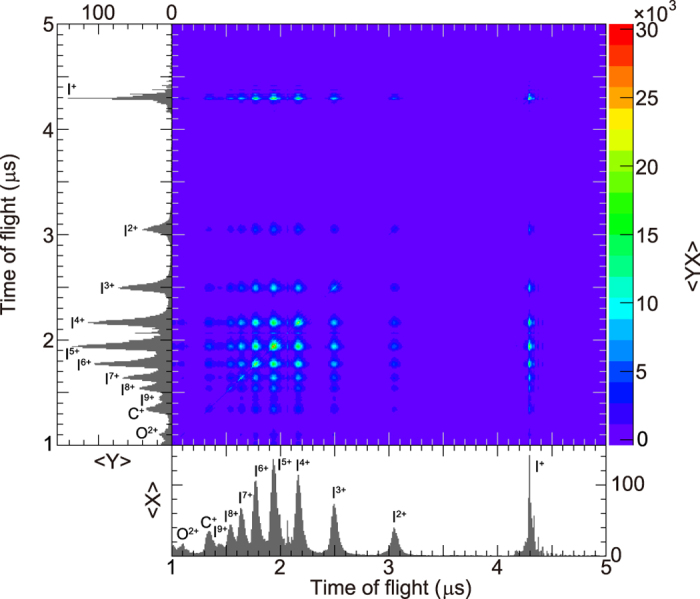
Covariance map of fragment ions. Spots on the diagonal line correspond to fragment-ion pairs with equal charges. Off-diagonal spots correspond to fragment-ion pairs with unequal charges. A small portion of I^+^ ion signal is created by the Nd:YAG laser.

Fragment ions are mainly produced in the following scenario: highly charged molecular ions are created via Auger cascades after I 2*p* photoionization by XFEL pulses, these create ion pairs of I^*m*+^ and I^*n*+^, which then dissociate back-to-back due to a Coulomb explosion between them. Figure 8 shows a covariance map of fragment ions for 2,500 XFEL pulses with the horizontally polarized Nd:YAG laser pulses. A small portion of the I^+^ ions is created by the Nd:YAG laser. This figure indicates that the main contribution to the prominent ring between *r* = 7.5 mm and 10.5 mm in Fig. 3(e) is due to the fragment ions with charge states of 4+ to 6+. For the fragment-ion pairs with the charge-separation combinations of (4 – 4), (4 – 5), (4 – 6), (5 – 5), (5 – 6), and (6 – 6), which produce images between *r* = 7.5 mm and 10.5 mm, a kinetic energy release was estimated using SIMION^34^. Here, the kinetic energy release is 56 eV for I^4+^ – I^4+^ pairs with an internuclear distance of 4.1 Å when the Coulomb explosion occurs after the cascade Auger decays and 130 eV for I^6+^ – I^6+^ pairs with a distance of 3.9 Å.

### Data processing for polar plots

**Figure 9:**
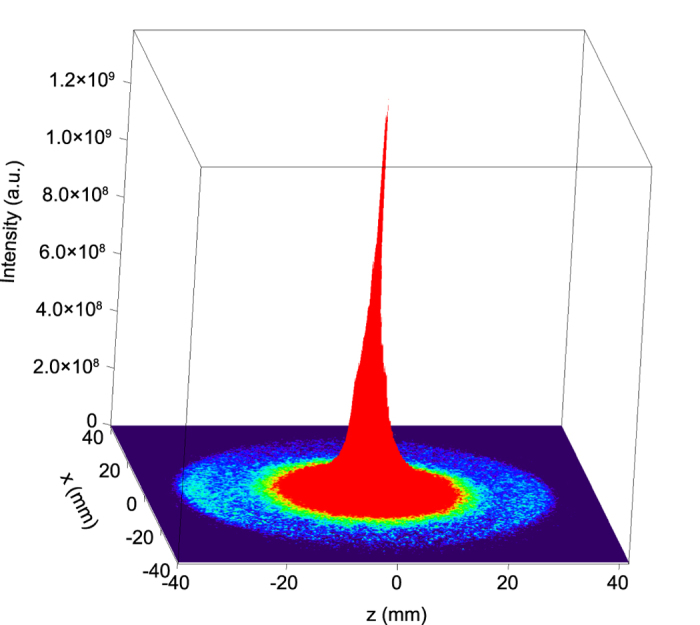
Three-dimensional representation of the electron image shown in Fig. 3(b). Compared to the outer-ring intensity for 2*p* photoelectrons, the intensity in the central region is extremely strong. The tail of the central region extends to the outer ring.

A three-dimensional representation of the 2D photoelectron image in Fig. 3(b) is shown in Fig. 9. As seen in this figure, the central image, because of low-energy electrons, has a sharp and strong peak at the center and a slightly asymmetric long tail that extends to the photoelectron outer ring. Accordingly, we subtracted the extrapolated asymmetric tail contribution from the signals in the range of *r* = 27.5 mm to *r* = 35 mm, sector-by-sector on the *xz* plane. Then, we integrated the signals over Δ*r* *=* (27.5‐35) mm, which corresponds to an azimuthal acceptance angle of 60^°^, and over a polar angle of 30^°^ on the *xz* plane. Finally, the left-side and right-side numerical data with respect to the *z* = 0 plane were averaged, and the upper and lower data with respect to the *x* = 0 plane were averaged, considering the symmetry restriction of the XPD pattern imposed by the experimental geometry, see Fig. 3. The large error bars of the data near the *x* axis in Fig. 4 are due to the subtraction of the background image (without sample gas) from the signal image (with sample gas).

It should be noted that the strong Nd:YAG laser field may give rise to a sideband structure in the 2*p* photoelectron spectrum as the result of above-threshold ionization^35^, in which photoelectrons interact with the laser field through the absorption or emission of a number of laser photons. In our experimental conditions, the number of exchanged laser photons is estimated to be ≤10 (Ref. 36). Thus, the sidebands may be included in our photoelectron images. However, these are not visible since they are smeared out by the photon-energy shot-by-shot fluctuation of 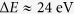.

### General formula of photoelectron angular distributions

The photoelectron angular distribution from a gas of isolated, randomly oriented molecules has the form


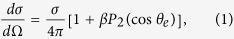


where σ is the integrated cross section, β is the asymmetry parameter, and *θ*_*e*_ is measured from the electric vector of the XFEL^37^,^38^. 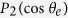 denotes the Legendre polynomial of the second order.

If, instead, the molecules have a definite orientation, then the angular distribution is described by an alternative form from Eq. (1). For example, when the molecular axis is parallel to the electric vector of the XFEL (see Fig. 3), the angular distribution in the *xz* plane can be expressed by


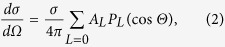


where the polar angle *Θ* is measured from the molecular *z* axis^39^,^40^. 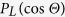 denotes the Legendre polynomial of the *L*th order, and the *A*_*L*_ coefficients are calculated from the relevant dipole-matrix elements. Due to the parity selection rule, the summation over *L* is restricted to even integers for molecules having inversion symmetry (like the I_2_ molecule). When the molecular axis is perpendicular to the electric vector of the XFEL (see Fig. 3), the angular distribution in the *xz* plane is written as


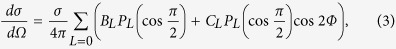


where the azimuthal angle *Φ* is measured from the molecular *x* axis^39^,^40^. Namely, the azimuthal-angle distribution is restricted by the conservation of the angular momentum component on the molecular axis for linear molecules. In contrast to this, the polar-angle distribution is determined by intramolecular photoelectron diffraction.

Parameterizations of Eqs (2) and (3) for the photoelectron angular distributions in the molecular frame, in other words the XPD, are derived from molecular photoionization theory. Numerical results of XPD patterns calculated by multiple scattering XPD theory have the same forms of Eqs (2) and (3), which imply molecular symmetry restrictions. The XPD patterns can be reproduced by both the photoionization and XPD theories, although they are based on different approximations.

Irrespective of the degree of alignment, the polar-angle distribution of the XPD pattern for linear molecules has the same form as Eq. (2), although values of the *A*_*L*_ coefficients depend on the degree of alignment. For the present case, the degree of alignment 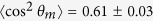 has been incorporated to reproduce our photoelectron diffraction data.

### Multiple-scattering X-ray photoelectron diffraction theory

A useful formula for X-ray photoelectron diffraction (XPD) amplitude *M*(***k***) for measuring photoelectron momentum ***k*** is written as


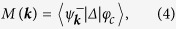


where 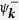 is the photoelectron wave function under the influence of optical potential in a system, *Δ* is the electron–photon interaction operator, and 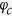 is a wave function of a core orbital localized on the site *A*. The site *A* also stands for the X-ray absorbing atom and the photoelectron emitter. By using the site-*t* matrix expansion of 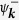
13,41,42, the amplitude can be written as multiple scattering series:


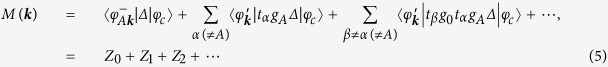


where 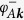 is the wave function of the photoelectron emitted from the X-ray absorbing atom *A* and 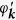 is the decaying plane wave under the influence of the imaginary part of the optical potential. Then, *g*_0_ is the decaying free Green’s function; *g*_*A*_ is the Green’s function influenced by the potential only on the absorbing atom *A* (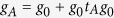); and *t*_*α*_ is the site-*t* matrix at the site α. As can be seen from Eq. 5, the first term, Z_0_, describes the direct photoemission amplitude without scattering from surrounding atoms, the second term, Z_1_, is then the single-scattering amplitude, and the third term, Z_2_, is the double-scattering amplitude, and so on. In this context, the XPD pattern is written as


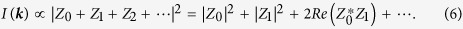


The third term, 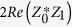, describes the interference effect between the direct and single-scattering waves, which is sensitive to molecular structure.

## Additional Information

**How to cite this article**: Nakajima, K. *et al.* Photoelectron diffraction from laser-aligned molecules with X-ray free-electron laser pulses. *Sci. Rep.* **5**, 14065; doi: 10.1038/srep14065 (2015).

## Figures and Tables

**Figure 1:**
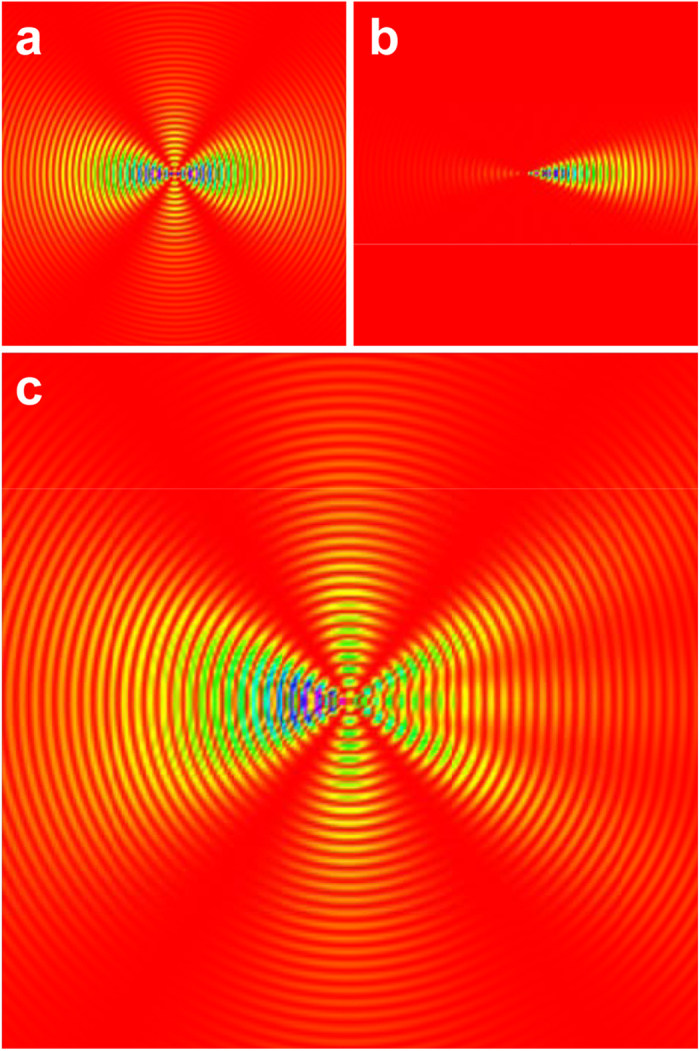
Illustration of X-ray photoelectron diffraction of a single aligned molecule. In a single aligned I_2_ molecule, a 2*p* photoelectron wave emitted from the left I atom (**a**) and a scattered wave by the right I atom (**b**) cause a fringe pattern due to interference between the two waves (**c**). The fringe pattern depends on an internuclear distance and photoelectron energy, although this simulation was done under the condition of an equilibrium internuclear distance and photoelectron energy of 140 eV.

**Table 1:** Minimum values of weighted sum of squared errors for the least squares fittings.

	**Data in** Fig. 4(a)	**Data in** Fig. 4(b)
Eq. (1) in Methods randomly oriented molecules	0.305	0.566
Eq. (2) in Methods aligned molecules	0.184	0.035

Compare the two numbers in each row. Comparison of the two numbers in each column does not make sense, see text.
